# Integrative analysis of transcriptome and metabolome reveals how ethylene increases natural rubber yield in *Hevea brasiliensis*

**DOI:** 10.3389/fpls.2024.1444693

**Published:** 2024-09-03

**Authors:** Hong Yang, Longjun Dai, Mingyang Liu, Xiaokang Fan, Liangruinan Lu, Bingbing Guo, Zhenhui Wang, Lifeng Wang

**Affiliations:** Rubber Research Institute, Key Laboratory of Biology and Genetic Resources of Rubber Tree, Ministry of Agriculture and Rural Affairs, Chinese Academy of Tropical Agricultural Sciences (CATAS), Haikou, China

**Keywords:** natural rubber, ethylene, latex yield, transcriptome, metabolome

## Abstract

*Hevea brasiliensis* is an important cash crop with the product named natural rubber (NR) for markets. Ethylene (ET) is the most effective yield stimulant in NR production but the molecular mechanism remains incomplete. Here, latex properties analysis, transcriptome analysis, and metabolic profiling were performed to investigate the mechanism of NR yield increase in four consecutive tappings after ET stimulation. The results revealed that sucrose and inorganic phosphate content correlated positively with dry-rubber yield and were induced upon ET stimulation. Stimulation with ET also led to significant changes in gene expression and metabolite content. Genes involved in phytohormone biosynthesis and general signal transduction as well as 51 transcription factors potentially involved in the ET response were also identified. Additionally, KEGG annotation of differentially accumulated metabolites suggested that metabolites involved in secondary metabolites, amino-acid biosynthesis, ABC transporters, and galactose metabolism were accumulated in response to ET. Integrative analysis of the data collected by transcriptomics and metabolomics identified those differentially expressed genes and differentially accumulated metabolites are mainly involved in amino-acid biosynthesis and carbohydrate metabolism. Correlation analysis of genes and metabolites showed a strong correlation between amino-acid biosynthesis during ET stimulation. These findings provide new insights into the molecular mechanism underlying the ET-induced increase in rubber yield and further our understanding of the regulatory mechanism of ethylene signaling in rubber biosynthesis.

## 1 Introduction

The rubber tree (*Hevea brasiliensis* Muell. Arg.) is cultivated in almost 30 tropical and subtropical countries to produce natural rubber (NR) (Qi et al., 2023). NR, the main constituent of which is cis-1,4-polyisoprene, is widely used owing to its unique characteristics, e.g. superior resilience, elasticity, efficient heat dispersion, abrasion resistance, and impact resistance (Zhang et al., 2023). Most importantly, it cannot be replaced by synthetic rubbers (Cornish, 2001). NR is obtained from the milky latex synthesized in highly specialized rubber-tree cells in the phloem, known as laticifers. The cytoplasmic content of these specialized laticifers is expelled in the form of a milky liquid when the bark is tapped, which is known as natural raw rubber latex (Adiwilaga and Kush, 1996; Qin et al., 2022). It is composed of water, rubber hydrocarbon, proteins, enzymes, nucleic acids, lipids, esters, and inorganic salts (Suksup et al., 2017). Rubber hydrocarbon, which accounts for 25% to 41% of the latex, is a polymer of isoprene units (Lehman et al., 2022). The other non-rubber substances also greatly affect the unique chemical and physical properties of NR (Lehman et al., 2022). Meanwhile, the capacity of rubber trees to regenerate latex components after tapping is a crucial factor that determines the efficiency of rubber yield (Zhu and Zhang, 2009). During the process of latex regeneration, the cells of the rubber tree undergo rapid metabolism to replenish the lost cytoplasmic contents. This process involves synthesizing and transporting large amounts of biomolecules, such as proteins, enzymes, nucleic acids, carbohydrates, lipids, and inorganic ions including potassium, inorganic phosphate, magnesium, etc.

The biosynthesis of NR, like many other secondary metabolites, is influenced by various phytohormones (Zhu and Zhang, 2009). As one of the major phytohormones, ET is widely used in agricultural production practices including natural raw latex production (Montoro et al., 2018; An et al., 2020; Fernández-Cancelo et al., 2022; Shu et al., 2023). ET releaser Ethephon or Ethrel are commonly used stimulators of rubber production worldwide and usually increase latex yield by 1.5- to 2-fold (Coupé and Chrestin, 1989; Zhu and Zhang, 2009). A substantial amount of research in recent decades has focused on deciphering the mechanism of ethylene action. Concerning the physiology and biochemistry aspects of rubber trees, ET affects the permeability of cellular membranes, leading to increased and prolonged latex flow and general regenerative metabolism (Coupé and Chrestin, 1989; Lu et al., 2023). As a side effect, ET treatment causes senescence of rubber tree seedlings, such as yellowing and abscised leaves (Nakano et al., 2021). Simultaneously, the enzyme activities of glutamine synthetase (GS) and chitinase are upregulated, and many metabolites accumulate in laticifers (Coupé and Chrestin, 1989; Amalou et al., 1991; Pujade-Renaud et al., 1994). Paradoxically, stimulation with ET does not affect the transcription level of certain genes involved in rubber biosynthesis or even downregulated (Chye et al., 1992; Sirinupong et al., 2005). Indeed, it is generally accepted that ET does not directly promote rubber biosynthesis, instead many antioxidant genes are upregulated upon exposure of rubber trees to ET. To reveal the molecular mechanisms underlying ET-induced stimulation of NR yield, much work has been performed with the latex or bark of rubber trees based on signal transcriptomics or proteomics analysis (Wang et al., 2015; Dai et al., 2016; Li et al., 2016; Nakano et al., 2021). Although these studies have identified numerous genes and proteins involved in response to ET, much information remains to be delineated to achieve a complete understanding of the mechanism by which ET improves NR production.

Owing to the complexity of the ET stimulation of rubber latex yield, a multi-omics analysis might help to uncover the mystery of ET in rubber trees. Therefore, we analyzed the physiological indicators of latex yield and generated metabolomes and transcriptomes for latex or bark from four consecutive tapping with or without ET stimulation. The results provide new insights into the molecular mechanism of the ET-induced increase in rubber yield.

## 2 Materials and methods

### 2.1 Plant materials and ET treatments

CATAS73397, an elite rubber tree cultivar was planted at the experimental farm of the Chinese Academy of Tropical Agricultural Sciences in Danzhou City, Hainan Province, (19°51’ 51N; 109°55’ 63E). All the trees were planted in 2007 and had their first tapping in 2014 with the latex harvesting system S/2 d3 (half-spiral tapping with a tapping frequency of 3 days), and trees with the same physical growth status were selected for treatment with ET. Before treatment, all trees were tapped to collect latex and bark samples for the negative control (ET0), and then the stimulation treatments were performed with 1.5% aqueous ethephon applied on the tapping panel by brush 3 days later. After 24 h, these trees were tapped to collect latex and bark samples (ET24, 24 h post-exposure to ET). After an additional 3 days, the trees were again tapped to collect latex and bark samples (ET1T, 96 h post-exposure to ET). After an additional 3 days, the trees were tapped again (ET2T, 168 h post-exposure to ET). All the treatments comprised three independent biological replicates, and every fifth tree served as a biological replicate. The latex samples were collected by a tumble placed on ice after tapping for the physiological character test and metabolomics analysis immediately, and the bark samples were collected and kept in liquid nitrogen for the following assays.

### 2.2 Latex yield and physiological indices test

Fresh latex (1 mL) was mixed with 9 mL of 2.5% trichloroacetic acid (TCA), and the mixture was centrifuged for 10 min at 4000 × *g*. The resultant rubber clump and TCA-treated serum were separated, and then the treated serum was used for further analysis after filtration through qualitative filter paper. Latex physiological index, i.e., dry-rubber content, sucrose content (mM), and inorganic phosphate content (mM) were measured following the method of Koryati and Elfiana (2019). Latex yield was converted into dry-rubber yield (gram tree^−1^ tap^−1^) to remove the effect of water contents.

### 2.3 Metabolite extraction and metabolomics analysis

Samples of fresh latex were centrifuged at 15,000 × g for 45 min at 4°C, and serum was collected for widely targeted metabolomics analysis. The extracted serum samples were sent to Shanghai Biotree Biotech for metabolomic sequencing and analysis. Briefly, SIMCA software (V16.0.2, Sartorius Stedim Data Analytics AB, Umea, Sweden) was used for the principal component analysis (PCA) and orthogonal projections to latent structures-discriminate analysis’ (OPLS-DA). Differentially accumulated metabolites (DAMs) were identified based on a Student’s t-test p-value of less than 0.05 and ‘variable importance in the projection’ of the OPLS-DA model greater than 1. DAMs were used to generate hierarchical clustering plots based on the Euclidean distance method, and complete linkage and heat maps were created using the Pheatmap package in R Studio. Additionally, commercial databases including KEGG (http://www.genome.jp/kegg/) and MetaboAnalyst (http://www.metaboanalyst.ca/) were used for pathway enrichment analysis (Kanehisa et al., 2015; Xia et al., 2015).

### 2.4 RNA preparation and transcriptome sequencing

The bark samples taken at ET0, ET24, ET1T, and ET2T were ground in liquid nitrogen, and total RNA was extracted according to Yang et al. (2020). Sangon Biotech Corporation (Shanghai, China) constructed the sequencing library and carried out Illumina sequencing. After filtering out reads containing the adaptor sequence and deleting low-quality reads, the clean reads were mapped to the *H. brasiliensis* genome using HISAT2.2.4 tools, and statistical analysis of the alignment results was conducted by RseQC (Wang et al., 2012; Kim et al., 2015). Differentially expressed genes (DEGs) were analyzed using the DESeq program of the R software package with parameters q-value < 0.05 and |log2 (fold change)| ≥ 1 (Love et al., 2014). Functional analysis of the obtained DEGs was performed per our previous study (Yang et al., 2020).

### 2.5 Quantitative PCR analysis

A total of 6 unigenes were selected to test the results of RNA-Seq by real-time quantitative PCR (RT-qPCR). *HbActin* was used as an internal control. All RT-qPCR primers were designed using Primer Premier 5 software (PREMIER Biosoft, Palo Alto, CA, USA) and synthesized by Sangon. Primer pairs are listed in **Supplementary Table 1**. RNA was reverse transcribed using the PrimeScript™ RT reagent kit with gDNA Eraser (Perfect Real Time; Takara, Da Lian, China). The amplified PCR products were monitored with the CFX96 Real-Time PCR detection System (Bio-Rad). All RT-qPCR experiments were carried out three times in independent runs for all references and selected genes. Ct values represent the mean ± SD of three biological replicates, and relative gene expression was evaluated using the 2^–ΔΔCt^ method (Livak and Schmittgen, 2001).

### 2.6 Statistical analysis

Statistical analysis was performed with SPSS statistics 27 (IBM Corporation, Armonk, NY, USA). One-way analysis of variance and the Student-Newman-Keuls test were performed for comparisons between multiple groups.

## 3 Results

### 3.1 Latex physiological changes of the trees under ET stimulation

**Figure 1:**
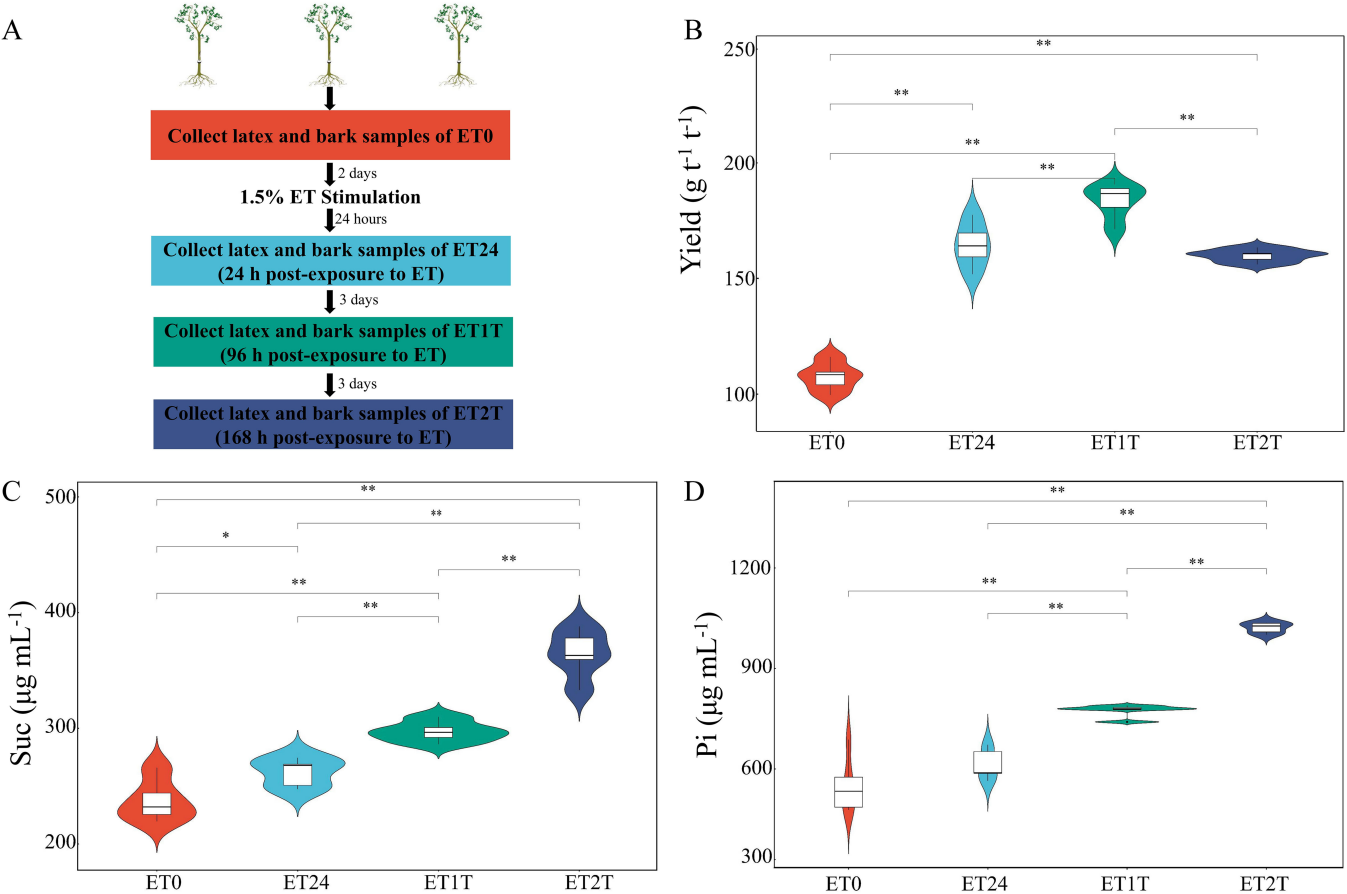
Experimental design and physiological indicators of rubber tree latex. **(A)** Experimental design for ET stimulation and sample collection. **(B)** The dry-rubber yield of rubber trees in response to ET. **(C)** The characteristics of sucrose (Suc) in rubber-tree latex during ET stimulation. **(D)** The characteristics of inorganic phosphate (Pi) in rubber-tree latex during ET stimulation. * indicated the significant difference at p<0.05 and ** indicated the significant difference at p<0.01.

The physiological parameters of latex usually reflect the latex metabolic status of rubber trees. To study how stimulation with ethephon affects these physiological parameters, we designed ET stimulation experiments and the dry-rubber yield and characteristics of physiological parameters such as sucrose and inorganic phosphate at multiple time points were evaluated (**Figure 1**). Compared with ET0 (before ET exposure, control), treatment with ET significantly increased the dry-rubber yield, especially at ET24 and ET1T (**Figure 1B**). The sucrose content increased continuously up to ET2T (**Figure 1C**). Slightly different from sucrose, a sustained increase of inorganic phosphate was observed starting at ET24 to ET2T (**Figure 1D**).

### 3.2 Identification of DEGs involved in the ET response

**Figure 2:**
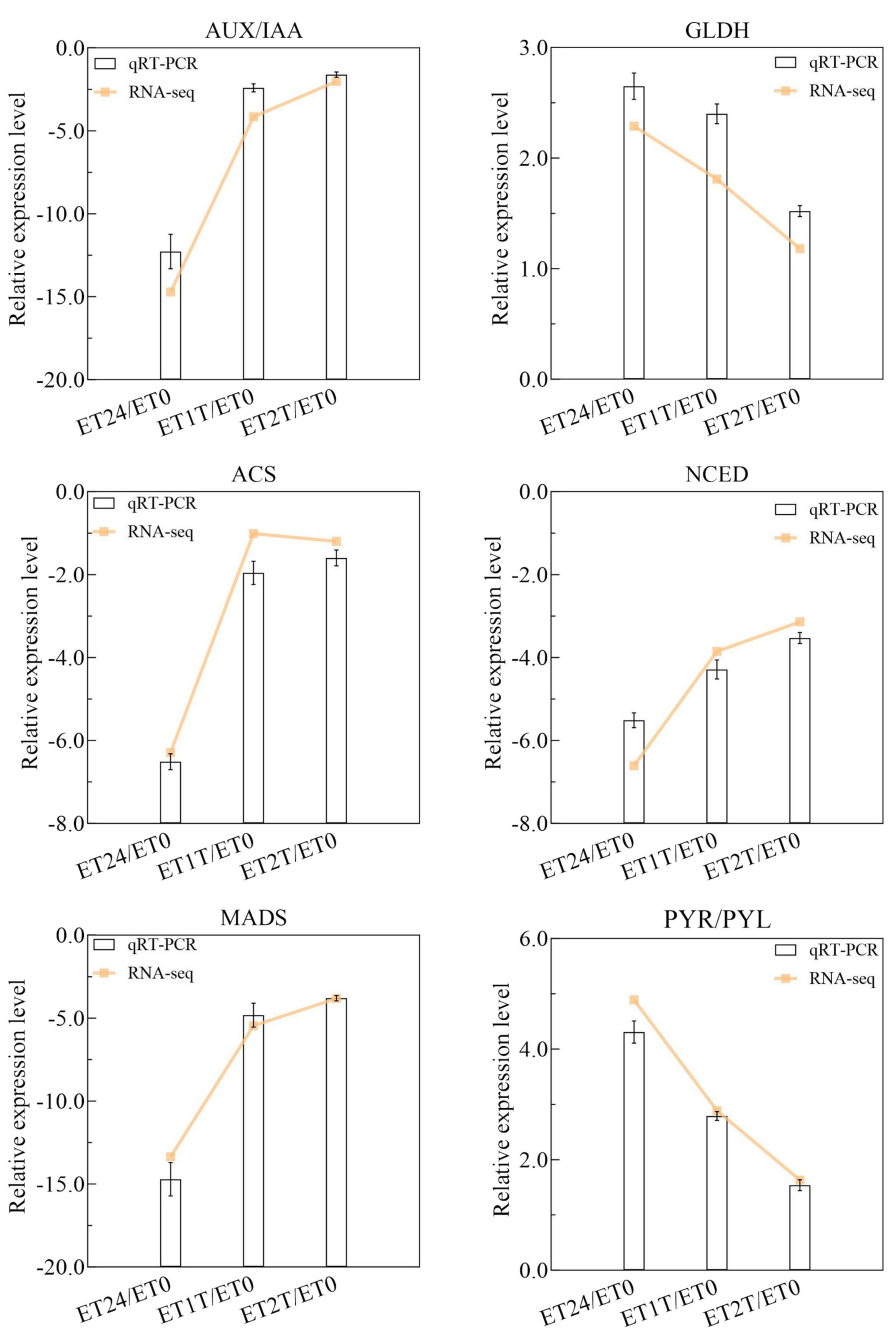
Relative expression level of six DEGs obtained by RT-qPCR and RNA-seq. The columns and lines represent log 2 (Fold change) determined by RT-qPCR and RNA-seq.

**Figure 3:**
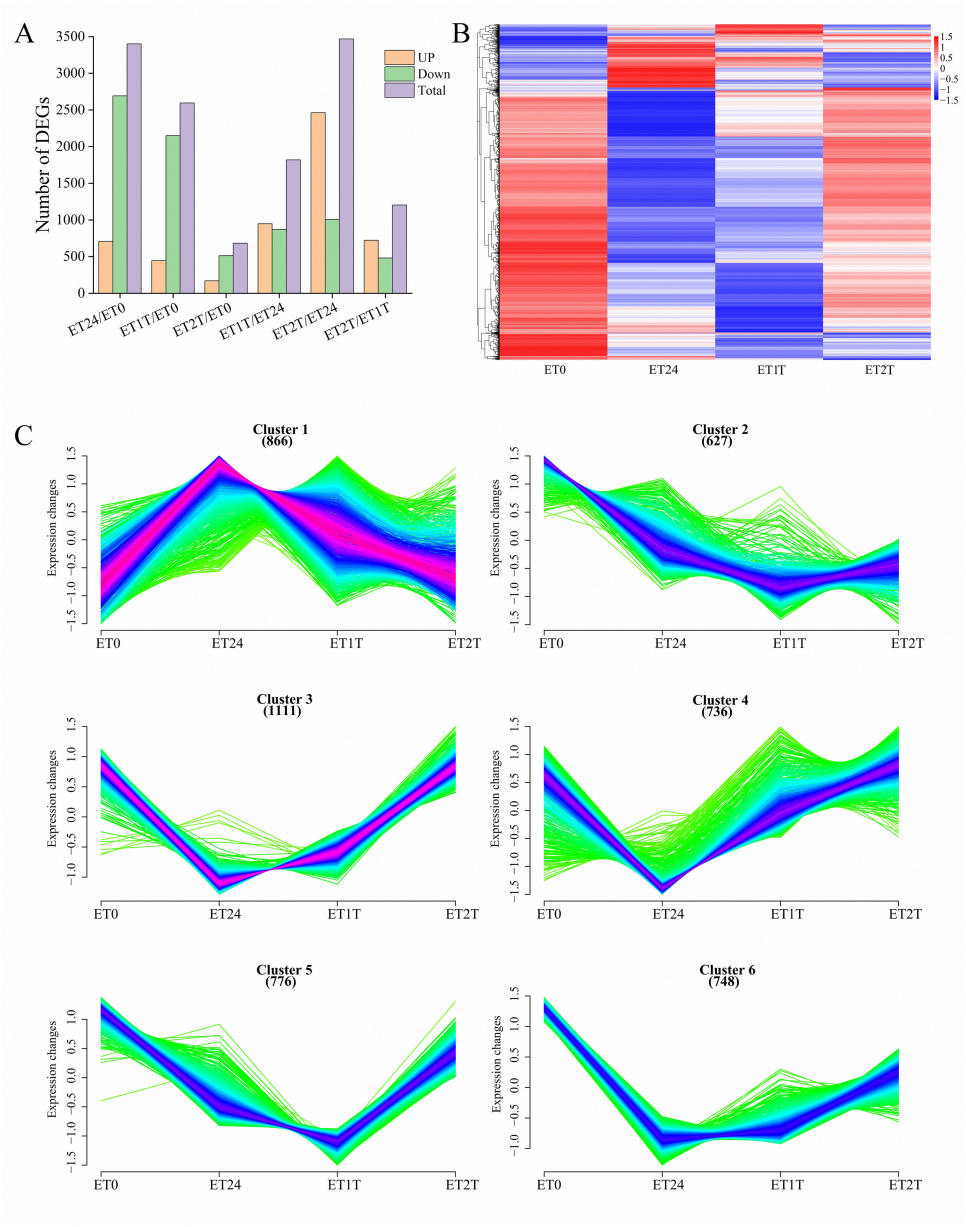
DEGs involved in the ET response. **(A)** Number of DEGs identified between different treatments of rubber trees (horizontal axis). Up, upregulated; Down, downregulated. **(B)** Heat map of DEGs identified upon stimulation with ET (ET0, control). **(C)** K-means cluster analysis of DEGs.

**Figure 4:**
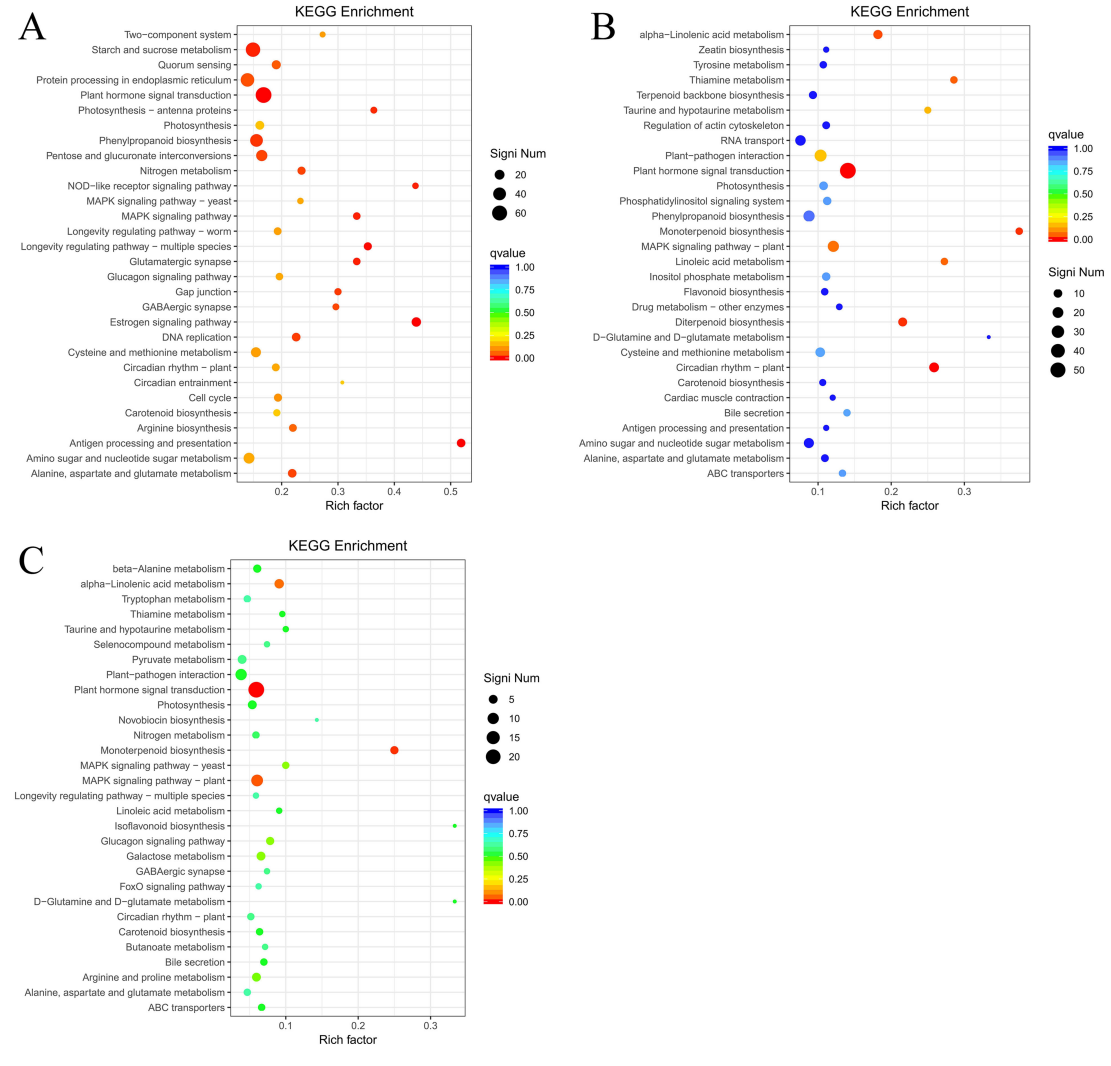
Analysis of KEGG terms enriched among the DEGs. The bubble diagram shows the significant enrichment KEGG pathways of the DEGs from ET24/ET0 **(A)**, ET1T/ET0 **(B)**, and ET2T/ET0 **(C)**.

To identify DEGs involved in the ET response of *H. brasiliensis*, rubber-tree bark was subjected to RNA sequencing (RNA-seq), and the raw data were deposited into the Genome Sequence Archive in the National Genomics Data Center, China National Center for Bioinformation, Chinese Academy of Sciences (accession number CRA012198). To verify the reliability of the RNA-seq data, six DEGs from different pathways were randomly selected for real-time quantitative PCR (RT-qPCR). The results showed that all the selected DEGs showed similar expression profiles in RNA-seq and RT-qPCR analyses, indicating that the RNA-seq data were accurate, credible, and highly reproducible (**Figure 2**). Further, the number of DEGs was compared between each treatment group. Compared with ET0, 3400 DEGs were identified for ET24 (709 upregulated, 2691 downregulated), 2595 for ET1T (445 upregulated, 2150 downregulated), and 683 for ET2T (170 upregulated, 513 downregulated) (**Figure 3A**; **Supplementary Table 2**). These results indicated that the transcription of most DEGs was suppressed by ET, especially at ET24 and ET1T (**Figures 3A, B**). Based on K-means cluster analysis of their expression patterns, the DEGs were divided into six clusters (**Figure 3C**). Compared with ET0, 866 DEGs in cluster 1 displayed a negative response to ET (**Figure 3C**). Moreover, 261 and 30 DEGs were downregulated or upregulated at all three time points relative to ET0 (**Supplementary Figure 1**; **Supplementary Table 3**). Among the common ET-upregulated DEGs, four (LOC110661531, LOC110638059, LOC110652110, LOC110668984) encode chitinase and one encodes pathogenesis-related protein 1 (LOC110662812) (**Supplementary Table 3**). KEGG analysis of DEGs from comparisons ET24/ET0, ET1T/ET0 and ET2T/ET0 revealed that most DEGs were enriched in the pathways for starch and sucrose metabolism (ko00500), phytohormone signal transduction (ko04075), and amino-acid metabolism (**Figure 4**).

### 3.3 Identification of phytohormone-signaling genes and transcription-factor genes among the candidate DEGs

**Figure 5:**
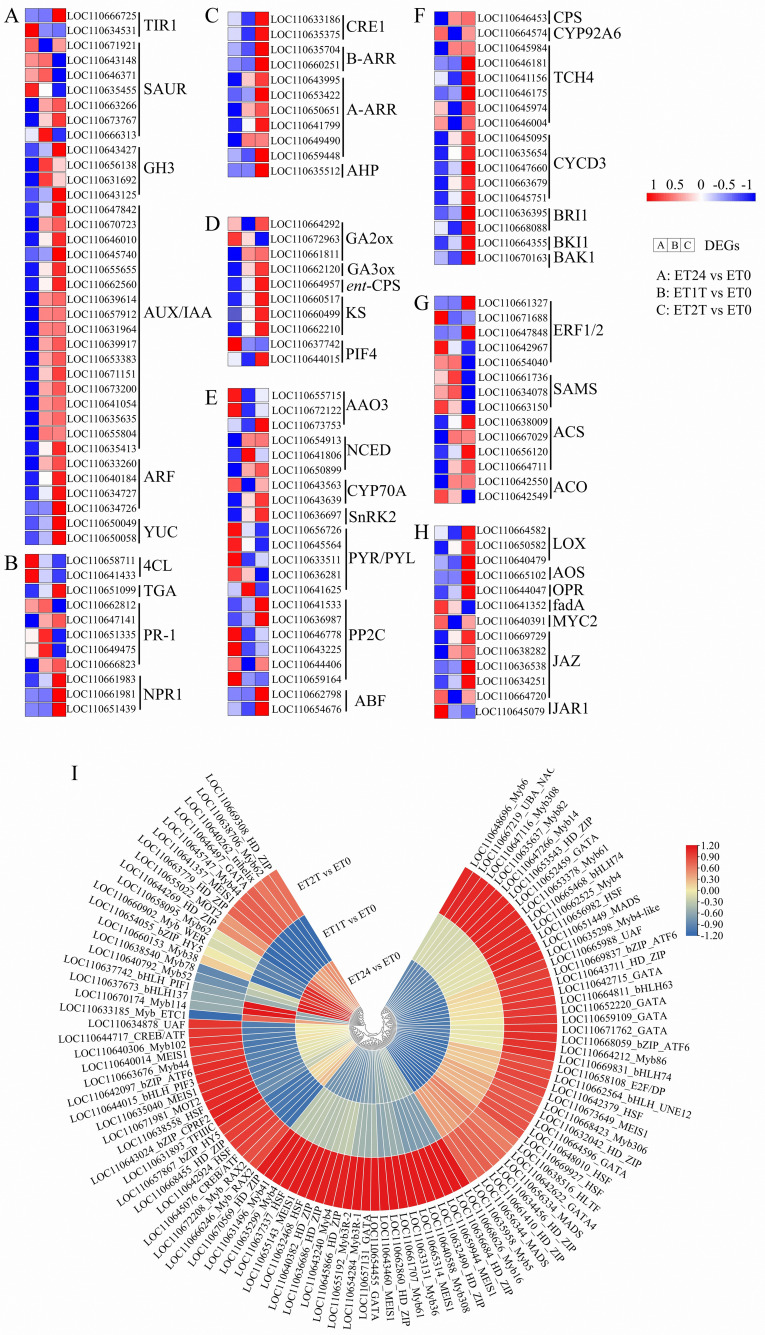
Phytohormone signaling and transcription factor genes involved in ET stimulation. Expression of genes involved in the biosynthesis of IAA **(A)**, SA **(B)**, CK **(C)**, GA **(D)**, ABA **(E)**, BR **(F)**, ET **(G)**, and JA **(H)** and heat map of transcription factors **(I)** involved in the ET response. The heat map was drawn with Tbtools software (Chen et al., 2020). Transcript level data from A to H were normalized using Z-score calculation. IAA, indole acetic acid; SA, salicylic acid; CK, cytokinin; GA, gibberellin; ABA, abscisic acid; BR, brassinosteroid; ET, ethylene; JA, jasmonic acid.

Exogenous ET application may alter and rebalance the cellular abundance of other major phytohormones such as auxin (indole acetic acid, IAA), cytokinin, gibberellin, abscisic acid, ET, brassinosteroid, jasmonate, and salicylic acid. To investigate the crosstalk between the ET signaling pathway and pathways governed by other phytohormones, the expression patterns involved in their biosynthesis and signal transduction pathways were analyzed (**Figures 5A–H**; **Supplementary Figure 2**). A total of 134 target genes were identified, including 36 involved in IAA signaling, 11 in salicylic acid and cytokinin signaling, 10 in gibberellin signaling, 22 in abscisic acid signaling, 17 in brassinosteroid signaling, 14 in ET signaling, and 13 in jasmonate signaling (**Figures 3A–H**; **Supplementary Table 4**). Based on expression relative to ET0, these genes were divided into four clusters (**Supplementary Figure 2**; **Supplementary Table 4**). The genes in cluster 1 were upregulated in response to ET at ET24, whereas these genes were downregulated at ET1T and ET2T, including one TIR1 and four SAUR for IAA, one GA2ox and one PIF4 for gibberellin, four PYR/PYL and one PP2C for abscisic acid, two ET response factors (ERF1/2), three S-adenosylmethionine synthases (SAMS) and one ACO for ET, one fadA and one JAR1 for jasmonate, and finally two 4CL and three PR-1 for salicylic acid (**Figure 5**; **Supplementary Figure 2**; **Supplementary Table 4**). The genes in clusters 2 and 4 were downregulated in response to ET at ET24, whereas these genes were upregulated at ET1T and ET2T, including one TIR1, two SAUR, four GH3, seventeen AUX/IAA, four ARF, and two YUC for IAA, two CRE1, two B-ARR, six A-ARR, and one AHP for cytokinin, two GA2ox and one PIF4 for gibberellin, one AAO3, three NCED, one CYP70A, one SnRK2, two PP2C, and two ABF for abscisic acid, two ERF1/2, four ACS, and one ACO for ET, one CPS, four TCH4, five CYCD3, two BRI1, one BKI1, and one BAK1 for brassinosteroid, three LOX, one AOS, one OPR, and four JAZ for jasmonate, and finally one TGA, two PR-1, and three NPR1 for salicylic acid (**Figure 5**; **Supplementary Figure 2**; **Supplementary Table 4**). The remaining genes were in cluster 3, in which genes were upregulated at ET24 and ET2T but downregulated at ET1T. Interestingly, the expression of two key ET biosynthesis genes (ACS and ACO) was suppressed by exogenous ET, whereas the expression of two downstream regulatory genes (EBF1/2 and ERF1/2) of the ET signaling pathway was increased in response to ET (**Figure 5**; **Supplementary Figure 2**; **Supplementary Table 4**).

To screen for transcription factors that are potentially involved in the ET response, we identified putative transcription factors among the DEGs, and a total of 101 transcription factors from 18 transcription-factor families were detected. The MYB superfamily was the largest member with 32 genes, followed by HD_ZIP with 16, GATA with 10, HSF and MEIS1 with 8, bHLH with 7, bZIP_ATF with 3, CREB/ATF with 2, UAF with 2, MOT2 with 2, and HLTF, TFIIIC, UBA_NAC, E2F/DP, bZIP_CPRF2, and trihelix with 1 (**Figure 5I**). With the parameters q-value < 0.05 and |log2 (fold change)| ≥ 2, 51 transcription factors were identified as major ET-responsive transcription factors, including 3 MADS, 14 MYB, 10 HD-ZIP, 5 HSF, 2 MEIS1, 1 HLTF, 3 bHLH, 7 GATA, 1 UAF, 1 E2F/DP, 1 bZIP_HY5, and 3 bZIP_ATF6 (**Supplementary Table 5**). These results implied that these genes likely participate in metabolic processes that increase rubber yield under ET stimulation.

### 3.4 Metabolomic response of rubber trees under ET stimulation

**Figure 6:**
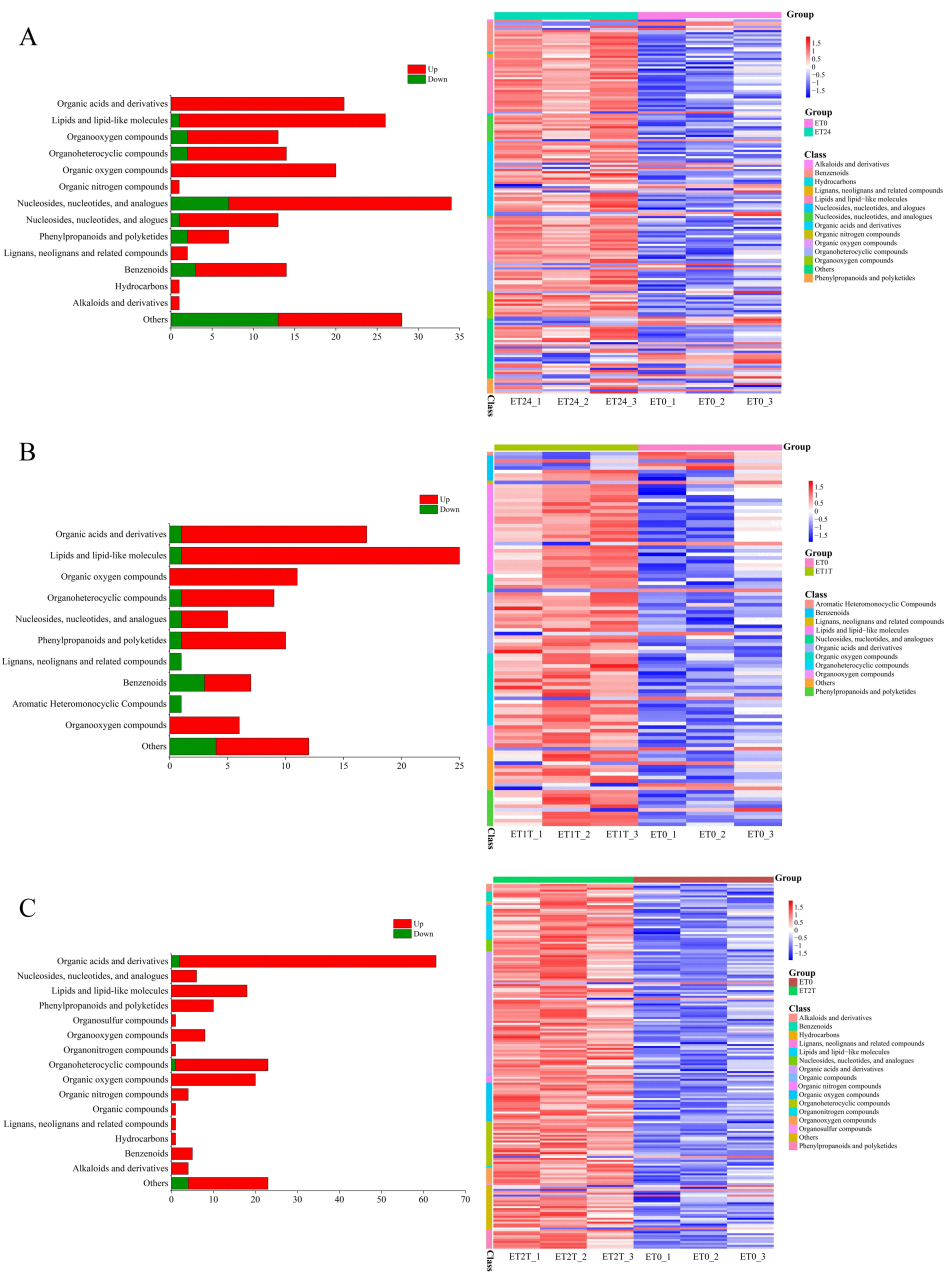
Number and heat map of DAMs involved in the ET response. The number of major classes of the detected metabolites and heat maps for ET24/ET0 **(A)**, ET1T/ET0 **(B)**, and ET2T/ET0 **(C)**.

Widely targeted metabolomics was performed to investigate changes in metabolites during ET treatment. Then, we facilitated statistical analysis of the number of common and unique DAMs in each contrast combination (**Figure 6**; **Supplementary Figure 3**). In the comparison combination of ET24/ET0, there were a total of 175 DAMs (**Supplementary Figure 3**). Similarly, there were 104 DAMs in the ET1T/ET0 comparison combination and 189 in the ET2T/ET0 comparison combination (**Supplementary Figure 3**). Additionally, there were 16 DAMs shared between ET24/ET0, ET1T/ET0 and ET2T/ET0. Furthermore, there were 39, 18, and 10 compounds shared between ET24/ET0 and ET1T/ET0, ET24/ET0 and ET24/ET0, ET1T/ET0 and ET2T/ET0, respectively (**Supplementary Figure 3**). The unique DAMs in the comparison combination of ET24 *vs* ET0, ET1T *vs *ET0 and ET2T *vs* ET0 were 102, 39, and 145, respectively (**Supplementary Figure 3**). To further analyze the composition and enrichment of DAMs in each contrast combination, we facilitated statistical analysis of the number of major classes of the detected metabolites and visualized by heat maps. For contrast combination ET24/ET0, the 175 DAMs were mainly enriched in nucleosides, nucleotides, lipids, organic acids, and organic oxygen compounds (**Figure 6A**). For contrast combination ET1T/ET0, the 104 DAMs were mainly enriched in lipids, organic oxygen compounds, and organic acids (**Figure 6B**). For contrast combination ET2T/ET0, the 189 DAMs were mainly enriched in organic acids (**Figure 6C**). These results indicated that the metabolic levels of organic acids and organic oxygen compounds might be highly relevant to the ET-induced stimulation of latex production in rubber trees.

We next carried out KEGG annotation of the DAMs identified at ET24/ET0, ET1T/ET0, and ET2T/ET0. For ET24, 50 pathways were identified, with notable enrichment in metabolic pathways (hbr01100), biosynthesis of secondary metabolites (hbr01110), biosynthesis of amino acids (hbr01230), ABC transporters (hbr02010), and galactose metabolism (hbr00052) (**Table 1**). ET1T had 21 pathways, with notable enrichment in metabolic pathways (hbr01100), biosynthesis of secondary metabolites (hbr01110), ABC transporters (hbr02010), biosynthesis of amino acids (hbr01230), and galactose metabolism (hbr00052) (**Table 1**). ET2T had 29 pathways, with notable enrichment in metabolic pathways (hbr01100), biosynthesis of secondary metabolites (hbr01110), ABC transporters (hbr02010), biosynthesis of amino acids (hbr01230), purine metabolism (hbr00230), and galactose metabolism (hbr00052) (**Table 1**).

**Table 1:** Key KEGG pathways in the metabolome.

Pathway ID	Description	Number of DAMs		
		ET24/ET0	ET1T/ET0	ET2T/ET0
hbr01100	Metabolic pathways	59	27	38
hbr01110	Biosynthesis of secondary metabolites	24	11	14
hbr01230	Biosynthesis of amino acids	14	6	7
hbr02010	ABC transporters	14	8	9
hbr00052	Galactose metabolism	9	5	4
hbr00230	Purine metabolism	5	–	6

-represents undetected.

### 3.5 Integrated analysis of the transcriptome and metabolome involved in the ET response of rubber trees

To investigate how treatment with ET increases latex yield, we performed an integrated analysis of the metabolome and transcriptome of ET-treated rubber trees. As shown in **Table 1**, amino-acid biosynthesis and carbohydrate metabolism were identified as the key pathways related to the ET-induced stimulation of latex production.

**Figure 7:**
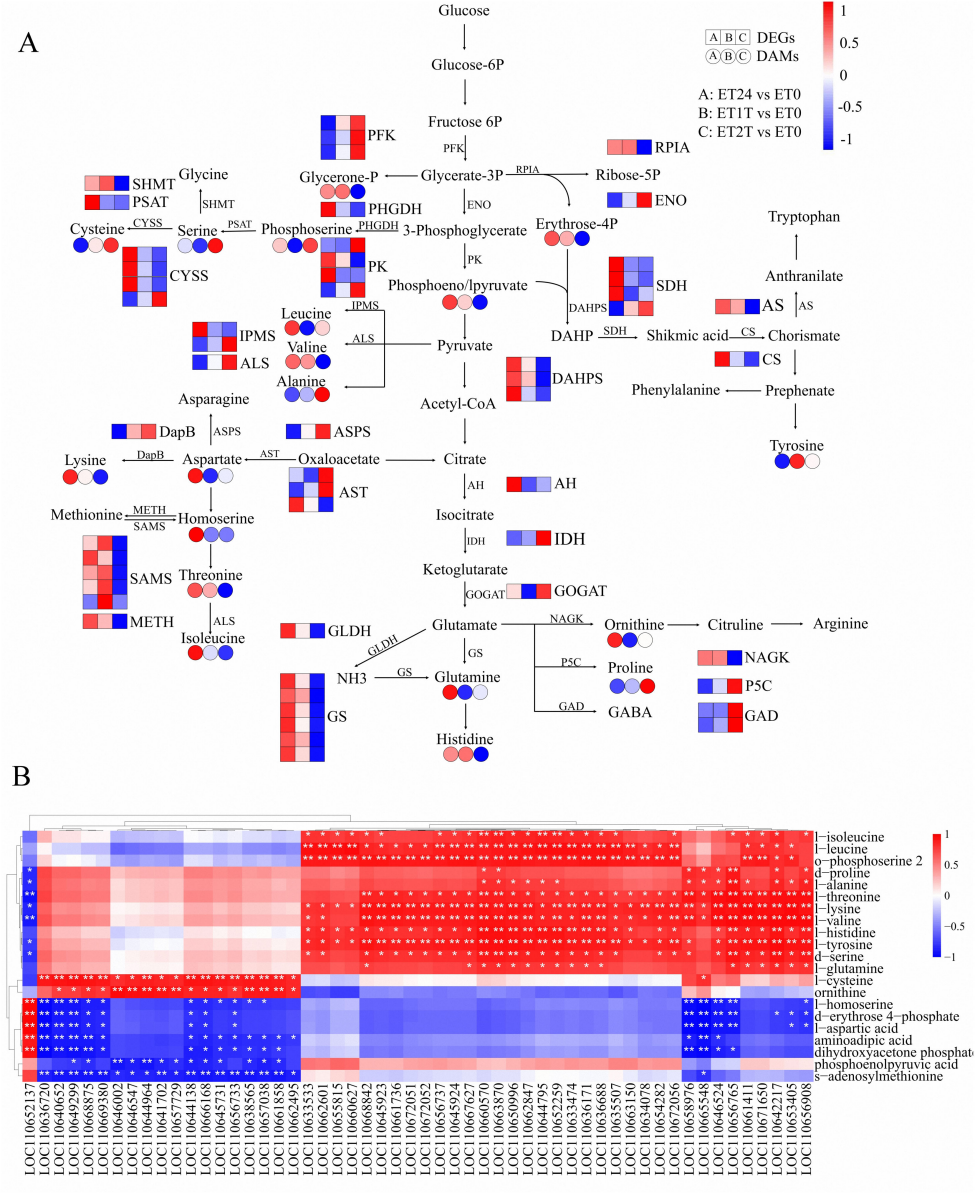
Expression profiles and Pearson correlation analysis of DEGs and DAMs relevant to amino-acid biosynthesis. **(A)** Schematic diagram of major genes and metabolites involved in amino-acid biosynthesis. Transcript level data were normalized using Z-score calculation. **(B)** Pearson correlation analysis between genes and metabolites involved in amino-acid biosynthesis. Genes that were significantly upregulated or downregulated in response to ET are indicated with *(p < 0.05) or **(p < 0.01).

The biosynthesis of amino acids is essential for plant growth and development, including responses to biotic and abiotic stresses (Zeier, 2013; Galili et al., 2016). At the metabolism level, 21 types of metabolites related to amino-acid biosynthesis were identified (**Figure 7**; **Supplementary Table 6**). For ET24/ET0, the following compounds were found to have accumulated: l-lysine, l-aspartic acid, phosphoenolpyruvic acid, l-ornithine, l-cysteine, dihydroxyacetone phosphate, l-leucine, l-histidine, l-valine, l-threonine, l-homoserine, d-erythrose 4-phosphate, and o-phosphoserine 2, whereas l-tyrosine and l-cysteine levels decreased (**Figure 7**). However, the abundance of most of these metabolites was decreased, except serine, cysteine, o-phosphoserine 2, alanine, and proline (**Figure 7**). Expression was altered for 54 genes related to amino-acid biosynthesis (**Figure 7**; **Supplementary Table 7**). The expression of 27 genes was significantly upregulated in response to ET signaling, especially early during stimulation (**Figure 7A**; **Supplementary Table 7**). The expression of 16 genes was significantly downregulated in response to ET signaling, especially early during stimulation (**Figure 7A**; **Supplementary Table 7**). Therefore, these genes and metabolites may be involved in the early ET response by regulating amino-acid biosynthesis. The results of the Pearson correlation analysis between the expression levels of the candidate genes and the accumulation of amino acids revealed that 23 genes were significantly positively correlated to at least 9 metabolites related to amino-acid biosynthesis (**Figure 7B**). Thus, these genes may play key roles in regulating amino-acid biosynthesis under ET stimulation in rubber trees.

**Figure 8:**
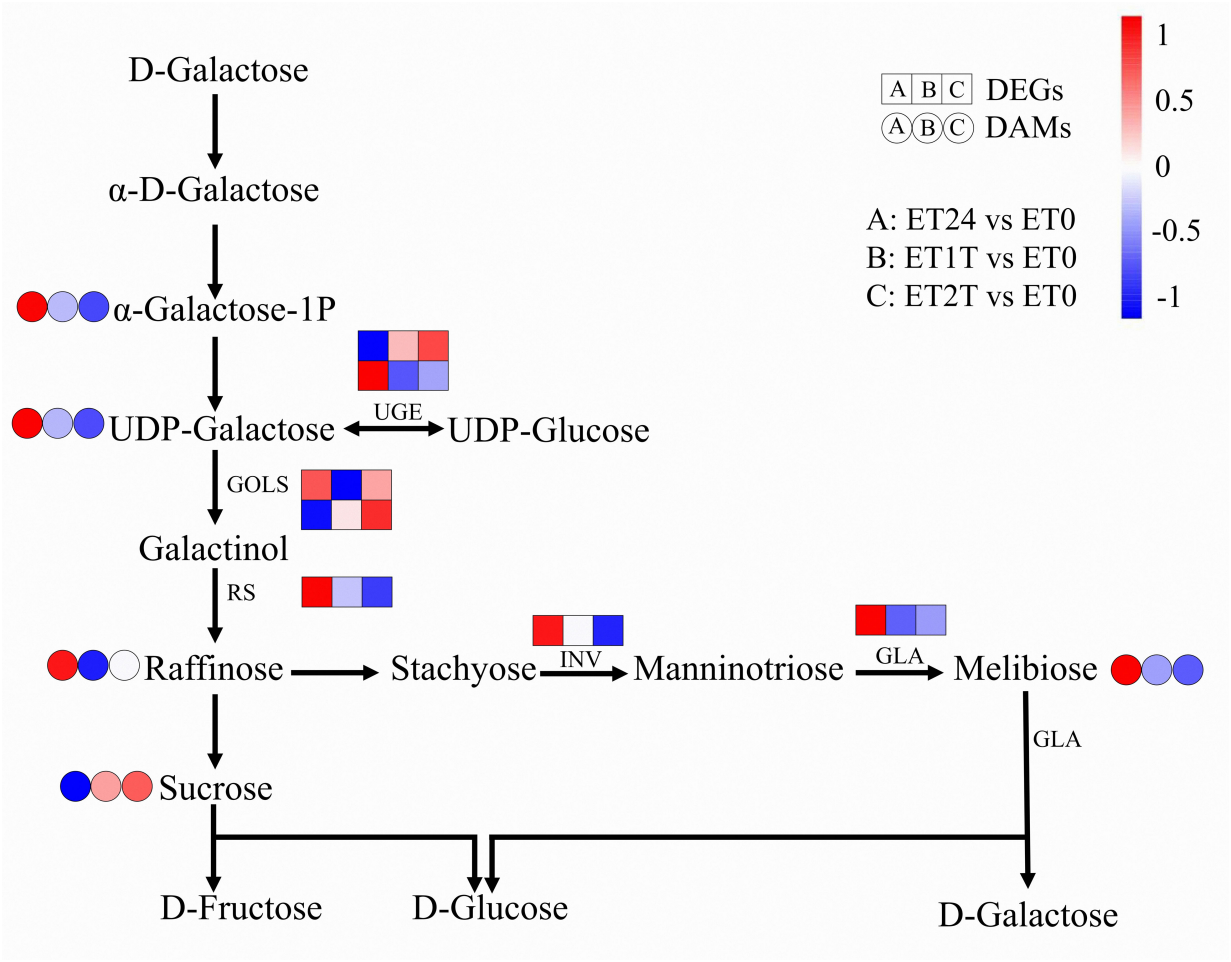
Expression profiles of DEGs and DAMs involved in carbohydrate metabolism. Transcript level data were normalized using Z-score calculation. GOLS, inositol 3-alpha-galactosyltransferase; UGE, UDP-glucose 4-epimerase; RS, raffinose synthase; GLA, alpha-galactosidase; INV, beta-fructofuranosidase.

Rubber biosynthesis requires a substantial amount of metabolic energy due to its high energy consumption, and carbohydrate metabolism provides energy and carbon skeletons (for use in biosynthesis) for this process. (Tang et al., 2010; Chao et al., 2017; Fang et al., 2021) At the metabolomic level, the content of each of raffinose, UDP-galactose, melibiose, and α-galactose 1-phosphate was increased, whereas the content of sucrose was decreased (**Figure 8**; **Supplementary Table 8**). However, the expression of seven DEGs related to carbohydrate metabolism was downregulated at ET24 and even more so at ET1T and ET2T (**Figure 8**; **Supplementary Table 9**).

## 4 Discussion

Identifying the key metabolic compounds that contribute to the ET-mediated rubber yield increase and revealing their underlying transcriptomic and transcriptional regulatory networks are important steps toward improving both NR yield and quality. Although much research has been based on the analysis of the transcriptome and/or proteome underlying the cellular signaling pathways of the latex or bark of rubber trees, the molecular mechanism of the ET-mediated enhancement of latex production and the effects of ET on NR quality remain a mystery. Here, through integrative analyses of metabolic and transcriptomic data across four consecutive tappings of rubber trees with or without ET treatment, we revealed the changes in global metabolites and genes that are regulated by ET.

### 4.1 ET-mediated rubber yield increase is a stress or defense response process

ET is a gaseous phytohormone that promotes fruit ripping, leaf senescence, abscission, and stress response in plants (Schaller, 2012; Iqbal et al., 2017; Gambhir et al., 2023). In many plants, the synthesis of ET is associated with biotic and abiotic stresses (Tao et al., 2015; Thao et al., 2015). In this respect, ET is often called “stress ET” (Bakshi et al., 2015). Over many decades of NR production, ET has been the most effective and commonly used enhancer of latex yield, but the adverse effects of ET should not be ignored, including tapping panel dryness (Osborne and Sargent, 1974). Like other latex-producing plants, the NR of the rubber tree is synthesized and stored in a specialized cellular organelle called the laticifer, which contributes to the defense strategy of plants (Konno, 2011; Hua et al., 2017). In laticifers, many latex proteins, such as peptidases, chitinases, lectins, pathogenesis-related proteins, and protease inhibitors, function as defense molecules to protect plants from stress-inducing agents such as fungi, insects, viruses, and wounding (Hua et al., 2017; Kitajima et al., 2018; Ramos et al., 2019). As such, in rubber trees, laticifers exude latex for defensive purposes, and humans have exploited the resultant increased turgor pressure by tapping trees. Our present results revealed that the expression of numerous genes was repressed by more than 75% in response to ET, e.g., 79% for ET24/ET0, 83% for ET1T/ET0, and 75% for ET2T/ET0 (**Figures 3A, B**). In contrast, the genes encoding chitinase and PR1 were oftentimes upregulated by ET (**Supplementary Table 3**), suggesting that ET constitutes a potent defensive signal. This may explain why overstimulation with ET or overtapping causes tapping panel dryness, a complex physiological response of the rubber tree that results in the cessation of latex flow during tapping.

### 4.2 Rubber yield increase under ET stimulation is a result of the cross-talk between different hormones

Endogenous phytohormone balance plays a vital role in latex regeneration and the duration of latex flow (Tungngoen et al., 2011; Chao et al., 2015; Deng et al., 2018; Montoro et al., 2018). However, how different phytohormones work together to regulate these two processes remains unclear. In our study, the biosynthesis and signaling pathways of ET and all the other phytohormones were particularly affected by ET (**Figure 5**; **Supplementary Table 4**). For ET biosynthesis, treatment with ET triggered the expression of SAMS, whereas the expression of ACS and ACO were repressed. Although the rubber-tree genome contains 181 genes encoding ERFs, only one *ERF* gene was dramatically upregulated. These results suggest that ET does not promote the biosynthesis of endogenous ET but rather ET signal transduction—especially at the early stage of ET stimulation. Similar results have been observed in the latex of rubber-tree clones PB206 and Reyan7-33-97 (Tang et al., 2016; Montoro et al., 2018). In addition, ET inhibits the expression of most genes related to phytohormone biosynthesis and signaling pathways. This suggests that the increase in yield stimulated by ET is the result of a combination of many phytohormones. Evidence reported by Tungngoen et al. (2011) revealed that auxin, salicylic acid, abscisic acid, and ET had identical effects on latex yield and latex total solid content in clone PB217. Together, research to date has demonstrated that treatment with ET increases latex yield by facilitating cross-talk between signaling pathways modulated by various phytohormones.

### 4.3 Amino-acid biosynthesis and carbohydrate metabolism involved in latex flow under ET stimulation

The effect of ET stimulation on amino-acid biosynthesis in rubber-tree latex remains unknown. Our results reveal for the first time the regulatory network of amino-acid biosynthesis in latex in response to ET stimulation using integrated metabolomic and transcriptomic analyses. The results show that ET altered the cellular content of each of the 21 amino acids or their derivatives, and 54 genes relevant to amino-acid biosynthesis were either upregulated or downregulated (**Figure 7**; **Supplementary Tables 6**, **7**). Pearson correlation analysis suggested that GS, PK, SAMS, SDH, DAHPS, CYSS, IPMS, METH, NAGK, PFK, PHGDH, PSAT, RPIA, and SHMT are the key genes controlling amino-acid biosynthesis under ET stimulation (**Figure 7B**). In rubber trees, NR biosynthesis requires a substantial amount of metabolic energy and induces a water deficit in laticifer cells owing to latex flow (Tungngoen et al., 2009; Tang et al., 2010). In all plants, i.e., including the rubber tree, raffinose-family oligosaccharides are critical for resistance to osmotic stress to assist in resistance to osmotic stress (Lintunen et al., 2016). In our present study, the cellular content of metabolites and altered expression of genes involved in the biosynthesis of raffinose-family oligosaccharides were altered by ET stimulation (**Figure 8**). Thus, the raffinose-family oligosaccharides would be involved in response to the water deficit regulation induced by ET stimulation during latex flow.

## 5 Conclusion

**Figure 9:**
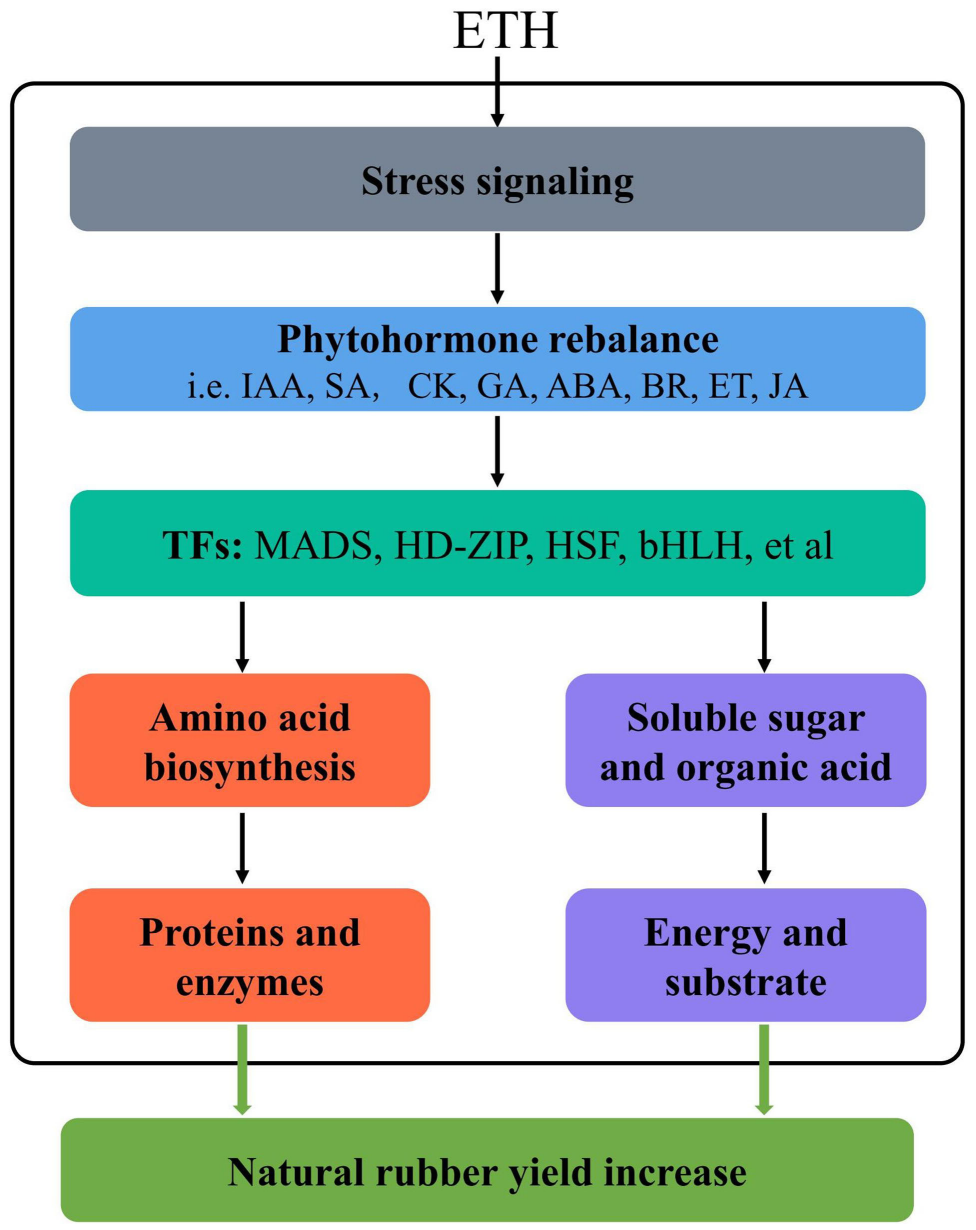
Putative model for ethylene-mediated natural rubber yield increase.

This study clarifies the molecular mechanisms of the ethylene-induced increase in latex yield in the rubber tree utilizing a multi-omics approach. We developed a potential underlying model based on our physiological, transcriptomic, and metabolomic analysis to elucidate the ethylene-mediated natural rubber yield increase (**Figure 9**). Briefly, exogenous ethylene acts as a stress signal and suppresses the expression of many genes related to primary metabolism by inducing endogenous hormone rebalance. The phytohormone then activates the expression of certain transcription factors, which regulate amino acid biosynthesis and carbohydrate metabolism. Amino acids provide sufficient proteins and enzymes for the NR biosynthesis process, which is essential for increasing rubber production. Ethylene promoted the biosynthesis of sucrose, galactose, raffinose and melibiose, indicating that these carbohydrates provide the rubber tree with high-energy substrates for NR biosynthesis under ethylene stimulation. These findings provide new insights into the molecular mechanism of the ethylene-induced increase in rubber yield.

## Data Availability

The datasets presented in this study can be found in online repositories. The names of the repository/repositories and accession number(s) can be found in the article/**Supplementary Material**.

## Supplementary material

Supplementary Figure 1 Venn diagram for comparison of the numbers of genes commonly downregulated (left) or upregulated (right) in response to ethylene.

Supplementary Figure 2 Cluster analysis of DEGs involved in phytohormone biosynthesis.

Supplementary Figure 3 Venn diagram of the number of DAMs.
