# Supplementary figures and images for: Integrative analysis of transcriptome and metabolome reveals how ethylene increases natural rubber yield in Hevea brasiliensis
Source: Front Plant Sci. 2024 Sep 3;15:1444693. doi: 10.3389/fpls.2024.1444693 (PMC11405334; doi:10.3389/fpls.2024.1444693)

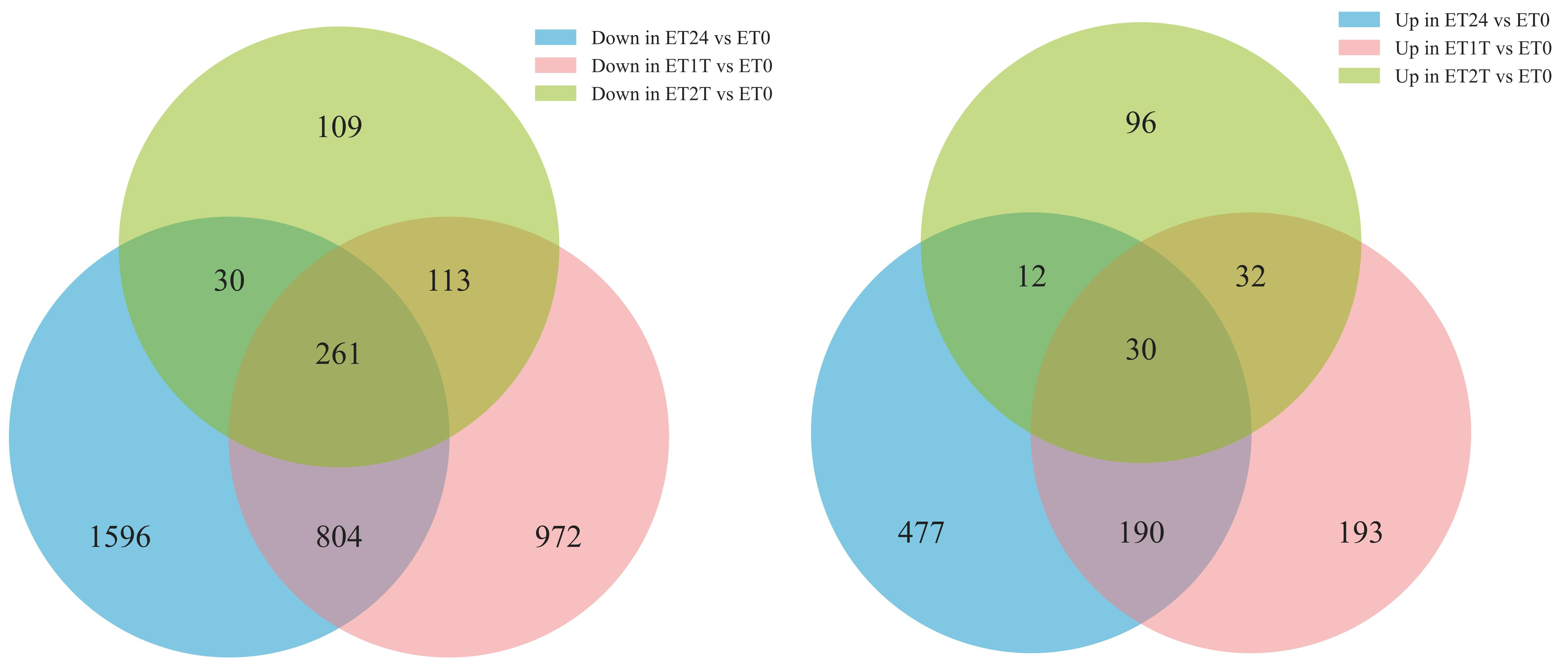

Supplement: Supplementary Figure 1 — Venn diagram for comparison of the numbers of genes commonly downregulated (left) or upregulated (right) in response to ethylene. [file Image1.jpeg]

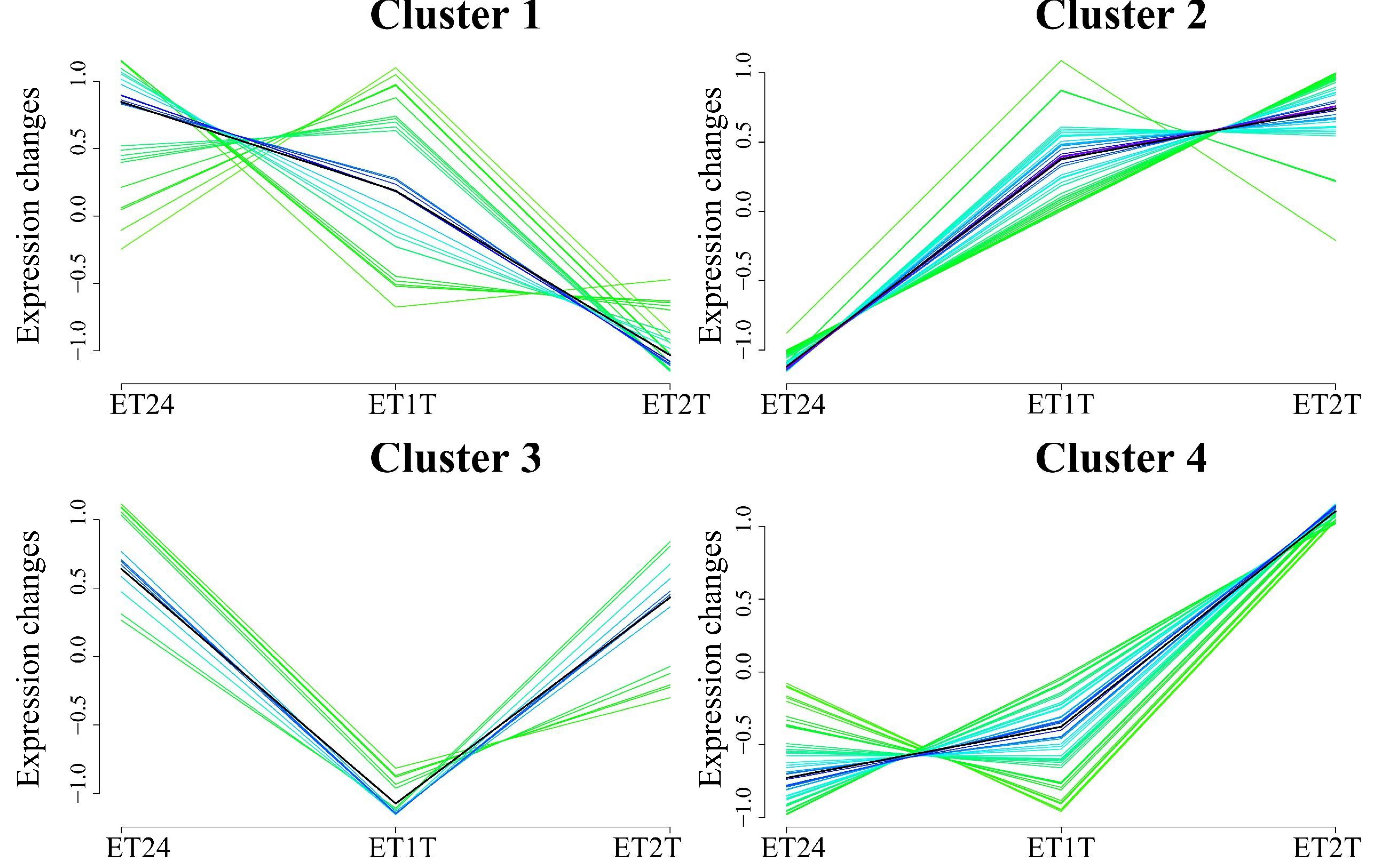

Supplement: Supplementary Figure 2 — Cluster analysis of DEGs involved in phytohormone biosynthesis. [file Image2.jpeg]

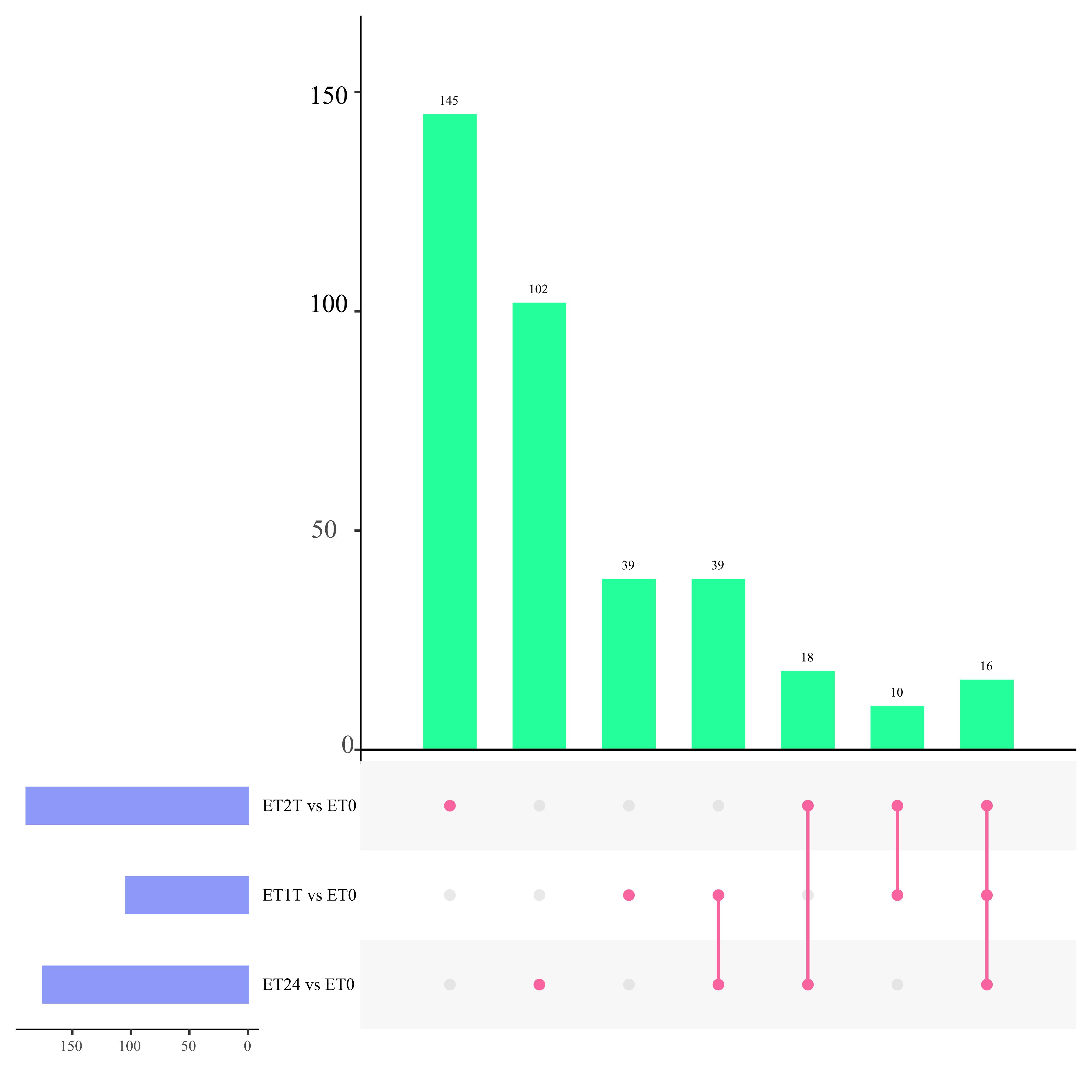

Supplement: Supplementary Figure 3 — Venn diagram of the number of DAMs. [file Image3.jpeg]
